# Improved overall survival is associated with adjuvant chemotherapy after definitive concurrent chemoradiotherapy for N3 nasopharyngeal cancer

**DOI:** 10.1038/s41598-022-16422-w

**Published:** 2022-08-04

**Authors:** Mu-Hung Tsai, Shang-Yin Wu, Hsi-Huei Lu, Tsung Yu, Sen-Tien Tsai, Yuan-Hua Wu

**Affiliations:** 1 Department of Radiation Oncology, National Cheng Kung University Hospital, College of Medicine, National Cheng Kung University, No. 138 Sheng Li Rd., Tainan, Taiwan; 2 Department of Oncology, National Cheng Kung University Hospital, College of Medicine, National Cheng Kung University, Tainan, Taiwan; 3 Division of Nuclear Medicine, Department of Medical Imaging, National Cheng Kung University Hospital, College of Medicine, National Cheng Kung University, Tainan, Taiwan; 4 Department of Public Health, College of Medicine, National Cheng Kung University, Tainan, Taiwan; 5 Department of Otolaryngology, National Cheng Kung University Hospital, College of Medicine, National Cheng Kung University, Tainan, Taiwan

**Keywords:** Head and neck cancer, Chemotherapy, Radiotherapy

## Abstract

Concurrent chemoradiotherapy is the established treatment for locally advanced nasopharyngeal carcinoma (NPC). However, there is no evidence supporting routine adjuvant chemotherapy. We aimed to demonstrate the effect of adjuvant chemotherapy on survival and distant metastasis in high-risk N3 NPC patients. We linked the Taiwan Cancer Registry and Cause of Death database to obtain data. Clinical N3 NPC patients were divided as those receiving definitive concurrent chemoradiotherapy (CCRT) with adjuvant 5-fluorouracil and platinum (PF) chemotherapy and those receiving no chemotherapy after CCRT. Patients receiving neoadjuvant chemotherapy were excluded. We compared overall survival, disease-free survival, local control, and distant metastasis in both groups using Cox proportional hazards regression analysis. Propensity-score matching was also performed to evaluate the independent effect of adjuvant PF in a matched cohort with similar baseline characteristics. We included 431 patients (152 and 279 patients in the adjuvant PF and observation groups, respectively). Median follow-up was 4.3 years. The 5-year overall survival were 69.1% and 57.4% in the adjuvant PF chemotherapy and observation groups, respectively (*p* = 0.02). Adjuvant PF chemotherapy was associated with a lower risk of death (hazard ratio [HR] 0.61, 95% confidence interval [CI] 0.43–0.84; *p* = 0.003), even after adjusting for baseline prognostic factors (HR 0.61, 95% CI 0.43–0.86; *p* = 0.005). Distant metastasis-free survival at 12 months was higher in the adjuvant PF chemotherapy group than in the observation group (98% vs 84.8%; *p* < 0.001). After adjusting for baseline prognostic factors, adjuvant PF chemotherapy was associated with freedom from distant metastasis (HR 0.11, 95% CI 0.02–0.46; *p* = 0.003). Adjuvant chemotherapy was also associated with a decreased risk of death (HR 0.59, 95% CI 0.41–0.85, *p* = 0.004) in a propensity score-matched cohort. Prospective evaluation of adjuvant PF chemotherapy in N3 NPC patients treated with definitive CCRT is warranted because adjuvant PF chemotherapy was associated with improved overall survival and decreased risk of distant metastasis.

## Introduction

Nasopharyngeal carcinoma (NPC) is a squamous cell carcinoma arising from the nasopharyngeal epithelium. It has a peculiar worldwide prevalence, with an age-standardised rate of 3/100,000 Southeast Asians and only 0.4/100,000 Caucasians^1^. Due to its unique location within the nasopharynx and high radiosensitivity, radiotherapy has traditionally been the cornerstone of curative treatment. The landmark Intergroup 0099 (INT-0099) trial established the role of chemoradiotherapy in locally advanced NPC, demonstrating improved overall survival (OS) with concurrent cisplatin followed by three courses of adjuvant cisplatin-fluorouracil (PF)^2^. This regimen is widely accepted as the standard of care, particularly in North America^3^.

However, managing compliance and toxicity associated with this regimen has always been challenging. Patients often experience severe side effects and exhibit poor nutritional status by the end of concurrent chemoradiotherapy (CCRT), and compliance with adjuvant chemotherapy is 50%-76% at best^1^. In one particular study, only 63% of patients assigned to the chemoradiotherapy arm could complete three cycles of adjuvant chemotherapy per protocol; improvements in cancer control were nullified by an increase in non-cancer-related deaths, resulting in similar OS^4^. Several subsequent trials chose to omit adjuvant chemotherapy in their design but were nevertheless successful in demonstrating the superiority of CCRT over radiotherapy alone in terms of OS, highlighting the pivotal role of CCRT^5–7^. Different meta-analyses have produced inconsistent results regarding the benefit of adjuvant chemotherapy in the setting of CCRT^8–11^. Current evidence-based guidelines recommend CCRT but are less vocal about the role of adjuvant chemotherapy owing to uncertain benefits and substantial toxicity^3,12^. This uncertainty is also reflected in the guideline published by the Taiwan Cooperative Oncology Group, which states the role of adjuvant chemotherapy after CCRT is still undefined^13^.

Adjuvant chemotherapy is based on preventing future disease recurrence by eradicating microscopic cancer cells. Therefore, its value is proportional to the risk of residual disease persisting after definitive treatment. In NPC, the N category is highly correlated with the risk of distant metastasis: nearly 50% of patients with N3 disease eventually develop distant metastasis^14^. Consequently, intensification of therapy is most likely to show benefit in this population. This risk-stratified approach is supported by retrospective reports in which the addition of adjuvant chemotherapy was associated with improved survival in the high-risk group but not in the low-risk group^15^.

Since N3 classification accounts for only 10–15% of all newly diagnosed NPCs, large-scale studies focusing on this subgroup are currently lacking^16^. In this study, we aimed to focus on the high-risk population of N3 patients and examine the effect of adjuvant chemotherapy on survival and metastasis. We hypothesised that adjuvant PF chemotherapy can improve survival and reduce the rate of distant metastasis in N3 NPC patients.

## Materials and methods

### Data sources

This study was conducted using nationwide data provided by the Health and Welfare Data Center (HWDC), established by the Ministry of Health and Welfare in Taiwan. The HWDC consolidates data gathered by the government from various sources, which is then de-identified and made available for research based on case-by-case approval^17^. Among the databases available in the HWDC, this study utilised three data sources: the National Health Insurance Research Database (NHIRD), which includes billing information on all National Health Insurance (NHI)-reimbursed examinations, medications, and treatments; the Taiwan Cancer Registry (TCR), which includes detailed staging and treatment information of cancer patients in Taiwan; and the Cause of Death database, which lists all death certificates issued in Taiwan. Reporting of NPC to the TCR started in 2009 with the long-form database, which included data on total radiation dose, modality, start and end dates of radiotherapy, timing of systemic and locoregional therapy (i.e. sequential or concurrent chemoradiotherapy) amongst other detailed information, requiring 114 fields in total for a single patient^18,19^. Notably, data on recurrence (including the date and site of recurrence) were also required elements, but updating beyond the initial registry entry was not mandatory. Nonetheless, quality assessments suggest that TCR ranks amongst the highest quality cancer registries not only in Asia but also worldwide^19^. All databases in the HWDC can be linked through a common but anonymised identifier. The latest edition of TCR available for analysis was 2015, while the latest edition of Cause of Death database was 2018.

This study received a certificate of exempt review from the Institutional Review Board of National Cheng Kung University Hospital. Requirement for informed consent was also waived. This research was performed in accordance with the Declaration of Helsinki.

### Study population

We selected patients aged 20 years and above with a diagnosis of NPC (ICD-O-3 site: C11) and with pathologically confirmed invasive carcinoma (ICD-O-3M-codes: 8010, 8020, 8070, 8071, 8072, and 8082). Our inclusion criteria required upfront CCRT of at least 60 Gray via intensity-modulated radiotherapy or volumetric-modulated arc therapy. Patients with prior malignancy, two-dimensional or three-dimensional conformal radiotherapy, or radiotherapy alone were excluded. Patients receiving neoadjuvant chemotherapy were also excluded.

### Patient covariates and outcome definition

We extracted data on age, sex, stage, Union for International Cancer Control/American Joint Committee on Cancer (UICC/AJCC) staging edition, treatment, and disease status at the last follow-up date from the TCR. Age was analysed as a continuous variable. Based on histopathological findings, we categorised the tumours as non-keratinising squamous cell carcinoma (ICD-O-3M-code: 8072), keratinising or unspecified squamous cell carcinoma (8070 or 8071), or other histologies including lymphoepithelial carcinoma, undifferentiated carcinoma, and unspecified carcinoma (8082, 8020, or 8010).

OS was calculated from the first day of radiotherapy to the day of death. The date of death was obtained from the Cause of Death database. Patients whose death records could not be found were considered alive and were censored on the last day of database records (31 December 2018). Disease-free survival (DFS) was defined as the time interval from the first day of radiotherapy to any recurrence; locoregional relapse-free survival (LRFS) and distant metastasis-free survival (DMFS) were defined as the time intervals from the first day of radiotherapy to locoregional or distant metastasis, respectively. DFS, LRFS, and DMFS were solely based on TCR data.

### Designation of adjuvant PF chemotherapy and observation groups

To confirm adjuvant chemotherapy status, we required double confirmation from both the TCR and NHIRD. The TCR indicated whether adjuvant chemotherapy was administered. We searched the linked NHI reimbursement database within the window period of 7–90 days from the last day of radiotherapy for prescription of the following cytotoxic drugs: cisplatin, carboplatin, 5-fluorouracil, tegafur-uracil, epirubicin, mitomycin-c, doxorubicin, and methotrexate. Patients with both registry-documented adjuvant chemotherapy and prescription of both 5-FU and one of cisplatin or carboplatin (i.e. the PF regimen) within this period were included in the adjuvant PF chemotherapy group; conversely, patients whose registry records indicated a lack of adjuvant chemotherapy, along with an absence of any cytotoxic drug prescription (as stated above), were included in the observation group. The reason we chose to require double confirmation is to reduce treatment heterogeneity as much as possible. Patients coded as receiving chemotherapy in the TCR but not prescribed PF possibly received an alternate chemotherapy regimen, such as cisplatin-gemcitabine (not reimbursed in Taiwan) or tegafur-uracil. Patients coded as not receiving chemotherapy but prescribed with cytotoxic drugs may have been coded with inaccurate information due to severe delay in adjuvant chemotherapy, treatment at a different institution, or because chemotherapy was prescribed for a second primary malignancy diagnosed during the NPC treatment course. In either case, a discordant registry and reimbursement data indicates treatment deviation from the typical adjuvant PF course, and we exclude these patients to reduce treatment heterogeneity.

### Statistical analysis

Baseline demographics and stage classification were compared using the chi-square test. Comparison of continuous variables was performed with the Kruskal–Wallis test or Student t-test.

We conducted univariate analysis by plotting Kaplan–Meier survival curves for previously defined endpoints and compared curves using the log-rank test. Multivariable Cox proportional hazards regression analysis was performed to estimate the independent effect of adjuvant chemotherapy.

Propensity score analysis was also conducted to assess the potential selection bias owing to imbalance of baseline factors resulting in decision to give adjuvant chemotherapy. The propensity score was created by fitting a multivariable logistic regression model including age, sex, histological subtype, clinical T classification, and N sub-classification; one-to-one matching was performed using nearest neighbour matching without replacement.

We performed landmark analyses to assess the effect of survival bias (immortal time bias). The typical adjuvant chemotherapy course is usually concluded within 6 months after the end of CCRT. Three separate analyses limited to patients surviving over 12, 18, and 24 months were performed.

All statistical analyses were conducted using SAS version 9.3 (SAS Institute, Cary, NC, USA), and R version 3.6.0 (R Foundation for Statistical Computing, Vienna, Austria). We calculated two-sided p-values with statistical significance defined at alpha = 0.05, along with 95% confidence intervals (CIs) to assess the precision of the estimates.

## Results

### Patient characteristics

Data of 10,231 patients diagnosed with NPC from 2009 to 2015 were retrieved from the TCR. All patients were staged according to the 7th edition of the UICC/AJCC staging system. We identified 431 patients after applying the inclusion and exclusion criteria (Supplementary Fig. 1). Of 431 patients, 152 (35.3%) patients were in the adjuvant PF chemotherapy group and 279 (64.7%) patients were in the observation group (Table 1). Approximately 80% of the patients were male, and nearly 80% had non-keratinising histology. There were roughly even numbers of T1, T2, T3, and T4 patients, and two-thirds of patients presented with N3b disease. Patients in the adjuvant PF chemotherapy group were younger (median age, 46 vs 50; *p* = 0.001) and less likely to have a non-keratinising histology (73% vs 82.4%; *p* = 0.04) than patients in the observation group. There was no significant difference in the distribution of T classification (*p* = 0.81) or N classification (*p* = 0.24) between these two groups. The median radiotherapy dose was 70 Gray in 35 fractions in the adjuvant PF group and 72 Gray in 36 fractions in the observation group (*p* = 0.23). Median follow-up was 4.3 years (range 0.17–9.83 years) for the entire cohort; median follow-up was 4.7 years in the adjuvant PF chemotherapy group and 4.0 years in the observation group (*p* = 0.11).

**Table 1:** Baseline patient and tumor characteristics (n = 431).

Characteristics	Adjuvant PF Chemotherapy (n = 152)	Observation (n = 279)	*p* value
**Sex**			0.26
Male	127 (83.6)	219 (78.5)	
Female	25 (16.4)	60 (21.5)	
**Age, median (IQR)**	46 (38.8–53)	50 (41–58)	0.001
**Age, mean (SD)**	45.8 (10.8)	49.6 (12.7)	0.002
**Histology**			0.04
Lymphoepithelial / undifferentiated / NOS carcinoma	38 (25.0)	42 (15.1)	
Squamous cell carcinoma, keratinizing or NOS	3 (2.0)	7 (2.5)	
Squamous cell carcinoma, non-keratinizing	111 (73.0)	230 (82.4)	
**Clinical T classification**			0.81
T1	51 (33.6)	106 (38.0)	
T2	30 (19.7)	51 (18.3)	
T3	34 (22.4)	55 (19.7)	
T4	37 (24.3)	67 (24.0)	
**Clinical N classification**			0.24
N3a	42 (27.6)	94 (33.7)	
N3b	110 (72.4)	185 (66.3)	
**Radiotherapy dose (Gray), median (IQR)**	70 (70–72)	72 (70–72)	0.23
**Radiotherapy fractions, median (IQR)**	35 (35–37)	36 (35–37)	0.83
**Median (IQR) follow-up, years**	4.7 (3.7–6.6)	4.0 (2.6–6.6)	0.11

IQR, interquartile range; SD, standard deviation; NOS, not otherwise specified.

### Predictors of OS

For the entire cohort, the 5-year OS rate was 61.6% (95% CI 57.1–66.6%). The OS rate decreased with progressive T-stage classification, with 66.5% for T1-2, 58.5% for T3, and 53.3% for T4 at 5 years. OS also decreased with N-stage classification (73.5% for N3a and 56% for N3b disease at 5 years).

**Figure 1:**
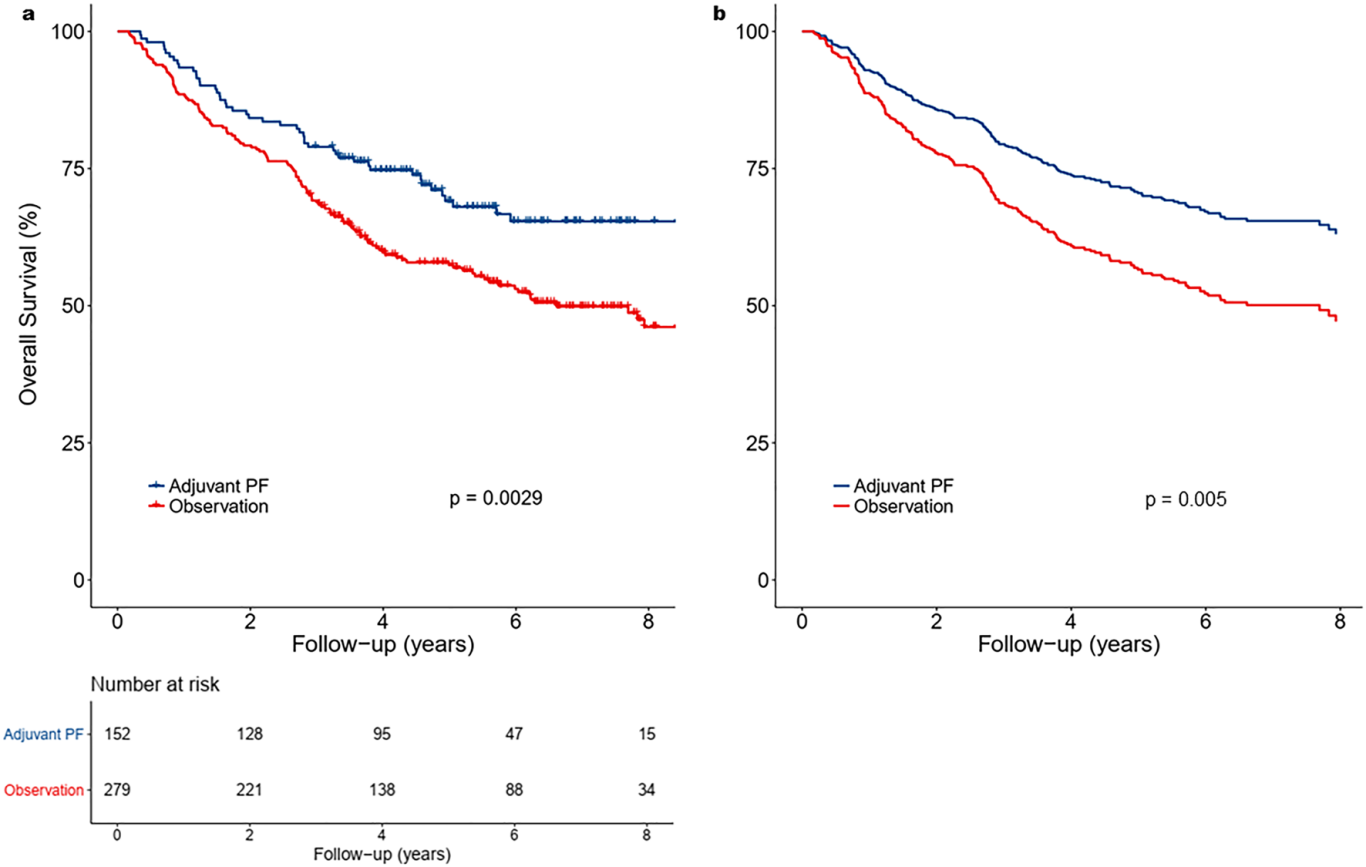
Kaplan–Meier survival curves (**a**) and multivariate adjusted survival curves (**b**) for overall survival show a higher survival rate in the adjuvant PF chemotherapy group. Figure created with R version 3.6.0 (https://www.r-project.org/).

On univariate analysis, older age, advanced T classification, and N3b disease were associated with an increased risk of death, whereas no effect was observed with sex or histology (Table 2). Adjuvant chemotherapy was associated with a lower risk of death (hazard ratio [HR] 0.61, 95% CI 0.43–0.84; *p* = 0.003). Patients in the adjuvant PF chemotherapy group had an improved OS (*p* = 0.003) (Fig. 1a) and a significantly higher 5-year survival rate than those in the observation group (69.1% vs 57.4%; *p* = 0.02). A multivariable Cox regression model adjusted for age, sex, T classification, N classification, and histology showed that adjuvant PF chemotherapy was independently associated with survival (HR 0.61, 95% CI 0.43–0.86; *p* = 0.005) (Fig. 1b, Table 2).

**Table 2:** Univariable and multivariable Cox proportional hazards model for overall survival (n = 431).

Variable	Univariable		Multivariable	
	Hazard ratio (95% CI)	*p* value	Hazard ratio (95% CI)	*p* value
**Age, continuous**	1.03 (1.01–1.04)	< 0.001	1.02 (1.01–1.04)	< 0.001
**Sex**				
Male	Reference		Reference	
Female	0.81 (0.55–1.20)	0.30	0.78 (0.53–1.16)	0.22
**Histology**				
Lymphoepithelial / undifferentiated / NOS carcinoma	0.78 (0.53–1.15)	0.21	0.94 (0.63–1.39)	0.74
Squamous cell carcinoma, keratinizing or NOS	0.87 (0.32–2.34)	0.78	0.84 (0.31–2.28)	0.73
Squamous cell carcinoma, non-keratinizing	Reference		Reference	
**Clinical T classification**				
T1	Reference		Reference	
T2	1.52 (0.98–2.37)	0.06	1.61 (1.04–2.50)	0.03
T3	1.63 (1.07–2.48)	0.02	1.72 (1.13–2.62)	0.01
T4	1.99 (1.36–2.93)	< 0.001	2.18 (1.48–3.22)	< 0.001
**Clinical N classification**				
N3a	Reference		Reference	
N3b	1.63 (1.16–2.29)	0.004	1.61 (1.14–2.28)	0.007
**Adjuvant treatment**				
Observation	Reference		Reference	
Adjuvant PF chemotherapy	0.61 (0.43–0.84)	0.003	0.61 (0.43–0.86)	0.005

CI, confidence interval; NOS, not otherwise specified.

Subgroup analysis suggested that the survival benefit of adjuvant PF chemotherapy was consistent across all subgroups (Supplementary Fig. 2). A test of interaction between these subgroups and the adjuvant PF chemotherapy on OS showed no evidence that the effect of chemotherapy is different among the subgroups.

### DFS, locoregional control, and distant metastasis

**Figure 2:**
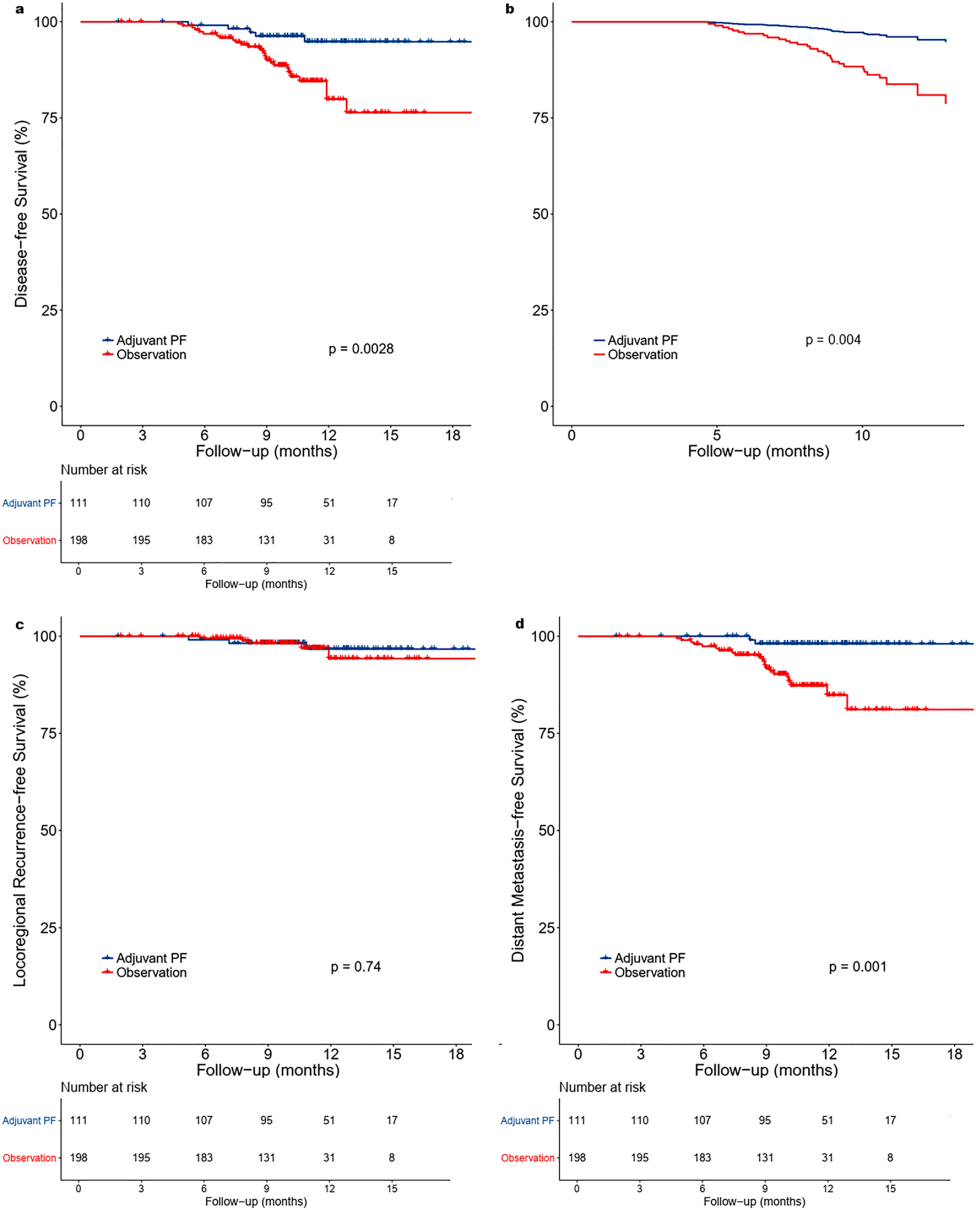
Kaplan–Meier survival curves (**a**) and multivariate adjusted survival curves (**b**) for disease-free survival show a higher percentage of patients being disease-free in the adjuvant PF chemotherapy group; meanwhile, Kaplan–Meier survival curves show (**c**) similar locoregional control and (**d**) higher rates of freedom from distant metastasis in the adjuvant PF chemotherapy group. Figure created with R version 3.6.0 (https://www.r-project.org/).

Among 431 patients, 310 cases (111 in adjuvant PF group, 199 in observation group) had recurrence data coded in the TCR. Median follow-up for recurrence endpoints was 10.7 months. We found a significant improvement in DFS in patients in the adjuvant PF chemotherapy group compared with those in the observation group (HR 0.25, 95% CI 0.10–0.67; *p* = 0.005) (Table 3). DFS at 12 months was 94.8% in the adjuvant PF chemotherapy group versus 79.9% in the observation group (*p* = 0.003) (Fig. 2a). The difference persisted after adjusting for age, sex, T classification, N classification, and histology (HR 0.24, 95% CI CI 0.09–0.63; *p* = 0.004) (Fig. 2b, Table 3).

**Table 3:** Univariable and multivariable Cox proportional hazards model for disease-free survival (n = 310).

Variable	Univariable		Multivariable	
	Hazard ratio (95% CI)	*p* value	Hazard ratio (95% CI)	*p* value
**Age, continuous**	0.99 (0.96–1.02)	0.62	0.98 (0.96–1.01)	0.26
**Sex**				
Male	Reference		Reference	
Female	1.84 (0.87–3.91)	0.11	1.64 (0.76–3.53)	0.21
**Histology**^**†**^				
Lymphoepithelial / undifferentiated / NOS carcinoma	0.72 (0.27–1.88)	0.51	0.84 (0.31–2.28)	0.74
Squamous cell carcinoma, non-keratinizing	Reference		Reference	
**Clinical T classification**				
T1	Reference		Reference	
T2	1.75 (0.72–4.29)	0.22	1.73 (0.70–4.27)	0.24
T3	1.13 (0.44–2.86)	0.80	1.25 (0.49–3.21)	0.64
T4	0.69 (0.22–2.13)	0.52	0.66 (0.21–2.06)	0.47
**Clinical N classification**				
N3a	Reference		Reference	
N3b	1.04 (0.49–2.20)	0.93	1.04 (0.48–2.28)	0.92
**Adjuvant treatment**				
Observation	Reference		Reference	
Adjuvant PF chemotherapy	0.25 (0.10–0.67)	0.005	0.24 (0.09–0.63)	0.004

CI, confidence interval; NOS, not otherwise specified.

^†^There were no events for the *Squamous cell carcinoma, keratinizing or NOS* group.

At 12 months, there was no difference in the locoregional failure rate between the adjuvant PF chemotherapy and observation groups (96.7% vs 94.2%; *p* = 0.49) (Fig. 2c). However, the possibility of DMFS at 12 months was higher in the adjuvant PF chemotherapy group than in the observation group (98% vs 84.8%; *p* < 0.001) (Fig. 2d). A multivariable Cox regression model adjusted for age, sex, T classification, N classification, and histology identified adjuvant PF chemotherapy as the only factor significantly associated with freedom from distant metastasis (HR 0.11, 95% CI 0.02–0.46; *p* = 0.003) (Supplementary Table 1).

### Propensity score-matched analysis

Propensity matching resulted in two groups of 152 patients each with balanced characteristics in age, sex, histological subtype, clinical T classification, and N sub-classification (Supplementary Table 2). In the propensity score-matched cohort, adjuvant chemotherapy was associated with a decreased risk of death (HR 0.59, 95% CI 0.41–0.85, P = 0.004) (Supplementary Fig. 3). Age, clinical T4 disease, and N3b classification were other factors associated with an elevated risk of death.

### Landmark analysis

Finally, we performed landmark analyses to restrict the analysis to patients surviving more than 12, 18, and 24 months, and the results were comparable with our primary analysis regarding OS (Supplementary Fig. 4).

## Discussion

This is the first registry-based study analysing high-risk N3 NPC patients treated with CCRT under contemporary techniques. In patients completing definitive CCRT, we observed that patients who received adjuvant PF chemotherapy had a higher chance of survival than patients who underwent observation alone, even after adjustment for known prognostic factors. Despite the short follow-up for recurrence endpoints, almost 15% of patients in the observation group developed distant metastasis after treatment, exhibiting the high risk of metastasis in this population. In contrast, an improved DFS and freedom from distant metastasis were observed in the adjuvant PF chemotherapy group. Receipt of adjuvant PF chemotherapy was associated with a remarkable 70% decrease in risk of any recurrence and 90% decrease in risk of distant metastasis. The different proportions of patients developing metastasis in the two groups suggest that reduction of distant metastasis is the main reason for the OS benefit associated with adjuvant PF chemotherapy. Landmark analysis suggested that our results were likely not impaired by immortal time bias, and it further implied that the benefits of adjuvant chemotherapy may persist for years beyond treatment.

The role of adjuvant chemotherapy in locally advanced NPC remains controversial. Four previous randomised trials failed to demonstrate the benefits of adjuvant chemotherapy^20–23^. Notably, although these trials aimed to enrol high- and intermediate-risk patients, clinical N3 patients comprised only 9–20% of the participants. The magnitude of benefit from adjuvant chemotherapy is likely smaller in intermediate-risk patients, which may explain the negative results of these trials. Consequently, the results of these trials are most applicable to intermediate-risk patients and may not be generalised to clinical N3 patients.

Conversely, several retrospective studies investigating clinical N3 patients have suggested that chemotherapy improves OS in this population^24,25^. Xu et al. reported outcomes of 140 patients with N3 NPC and revealed that adjuvant chemotherapy decreased the risk of death by up to 60%, while decreasing the risk of metastasis by 59%^25^. This relative risk reduction parallels our study, which showed a 40% decrease in risk of death and 90% decrease in risk of metastasis. To the best of our knowledge, our study represents the largest cohort till date to focus solely on N3 disease.

Neoadjuvant chemotherapy has emerged as a new treatment option in recent years based on two randomised controlled trials that showed OS benefits with neoadjuvant chemotherapy followed by CCRT compared to that with CCRT alone^26,27^. The appeal of neoadjuvant chemotherapy lies in the possibility of tumour volume reduction, leading to a reduction in radiotherapy volume. However, recovery from neoadjuvant treatment-associated toxicity may cause a delay in the initiation of definitive radiotherapy, resulting in an elevated risk of metastasis and death^28^. The NPC-0501 trial evaluated the effect of an induction-concurrent chemotherapy sequence compared to the traditional concurrent-adjuvant sequence in locally advanced nasopharyngeal carcinoma^29^. While no definitive conclusions could be drawn from the overall comparison, a secondary analysis suggested a potential improvement in progression-free survival in the induction-concurrent chemotherapy arm for patients receiving conventional fractionated radiotherapy. A network meta-analysis on chemotherapy for nasopharyngeal carcinoma concluded that the addition of either induction therapy or adjuvant therapy resulted in improved disease control to CCRT; however, the optimal choice between induction and adjuvant chemotherapy remains unclear^11^.

The current study had several limitations. Since there is no national consensus, the prescription of adjuvant chemotherapy is based on individual physician preferences and their assessment of individual patients, and therefore exists a likelihood of selection bias and/or residual confounding factors. Data on promising potential prognostic factors, such as plasma Epstein–Barr viral DNA, were not available in the TCR for analysis. Because we adopted a strict inclusion criteria requiring double confirmation from TCR and NHIRD, this study could not examine alternative chemotherapy agents such as gemcitabine^26^, docetaxel^30^, or lobaplatin^31^, which may have introduced additional selection bias; however, we believe this approach instils confidence in the homogeneity of adjuvant treatment in our patient population. Due to the nature of this study, toxicity and compliance of the treatment could not be evaluated. It is likely that not every patient in the adjuvant PF chemotherapy group received all three cycles of chemotherapy; however, this should bias results towards equivalence between observation and adjuvant chemotherapy. Given that updates beyond initial registry entry were not mandatory, there were high rates of missing recurrence status in the registry; therefore, it is important to consider the results on DFS, local recurrence, and distant metastasis as exploratory in nature. Despite these limitations, the strength of our study is the use of prospectively collected population-wide registry data, which genuinely reflect real-world patient population without excluding the elderly or patients with pre-existing comorbidities. Cross-linkage with additional databases, such as the Cause of Death database, allowed us to examine survival status with adequate follow-up.

In conclusion, we conducted this nationwide registry-based analysis of N3 NPC patients who were treated with upfront contemporary CCRT. Adjuvant chemotherapy was associated with improved OS and decreased risk of distant metastasis. Our results suggest that prospective evaluation of adjuvant PF chemotherapy in N3 NPC patients treated with definitive CCRT is warranted.

## Supplementary Information

Supplementary Information.

## Data Availability

The datasets generated and analysed during the current study cannot be made publicly available under the usage terms of the HWDC.
