# Supplementary material for: Improved overall survival is associated with adjuvant chemotherapy after definitive concurrent chemoradiotherapy for N3 nasopharyngeal cancer
Source: Sci Rep. 2022 Aug 4;12:13390. doi: 10.1038/s41598-022-16422-w (PMC9352661; doi:10.1038/s41598-022-16422-w)
Supplement: Supplementary file 1 — Supplementary Information. [file 41598_2022_16422_MOESM1_ESM.docx]

**Improved overall survival is associated with adjuvant chemotherapy after definitive concurrent chemoradiotherapy for N3 nasopharyngeal cancer**

**Supplementary Figure 1** Selection of study cohort. Abbreviations: IMRT, intensity-modulated radiotherapy; VMAT, volumetric modulated arc therapy; CCRT, concurrent chemoradiotherapy; TCR, Taiwan Cancer Registry; NHI, National Health Insurance; PF, platinum-fluorouracil regimen.

**
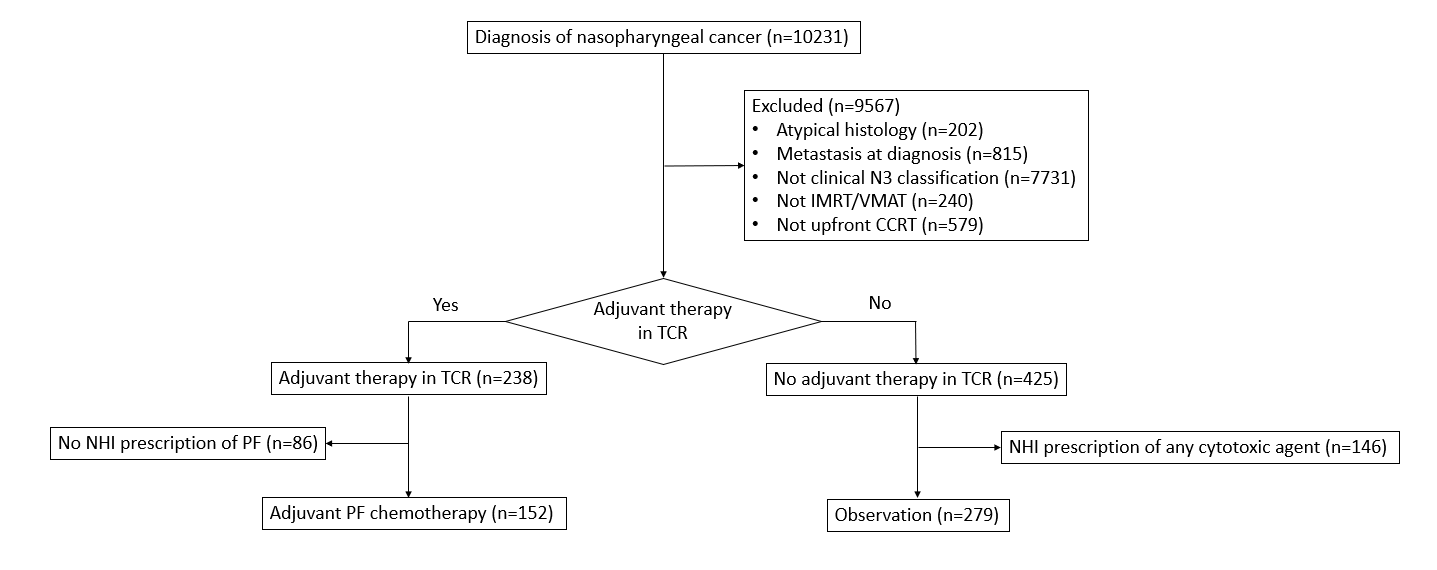
**

**Supplementary Figure 2** Forest plot of subgroup analysis on effect of adjuvant PF chemotherapy shows a consistent improvement in survival in all subgroups. *P*-values are shown for tests of interaction between the effect of adjuvant PF chemotherapy and given covariates. No significant interaction between subgroups and adjuvant chemotherapy was found. Figure created with R version 3.6.0 (https://www.r-project.org/).


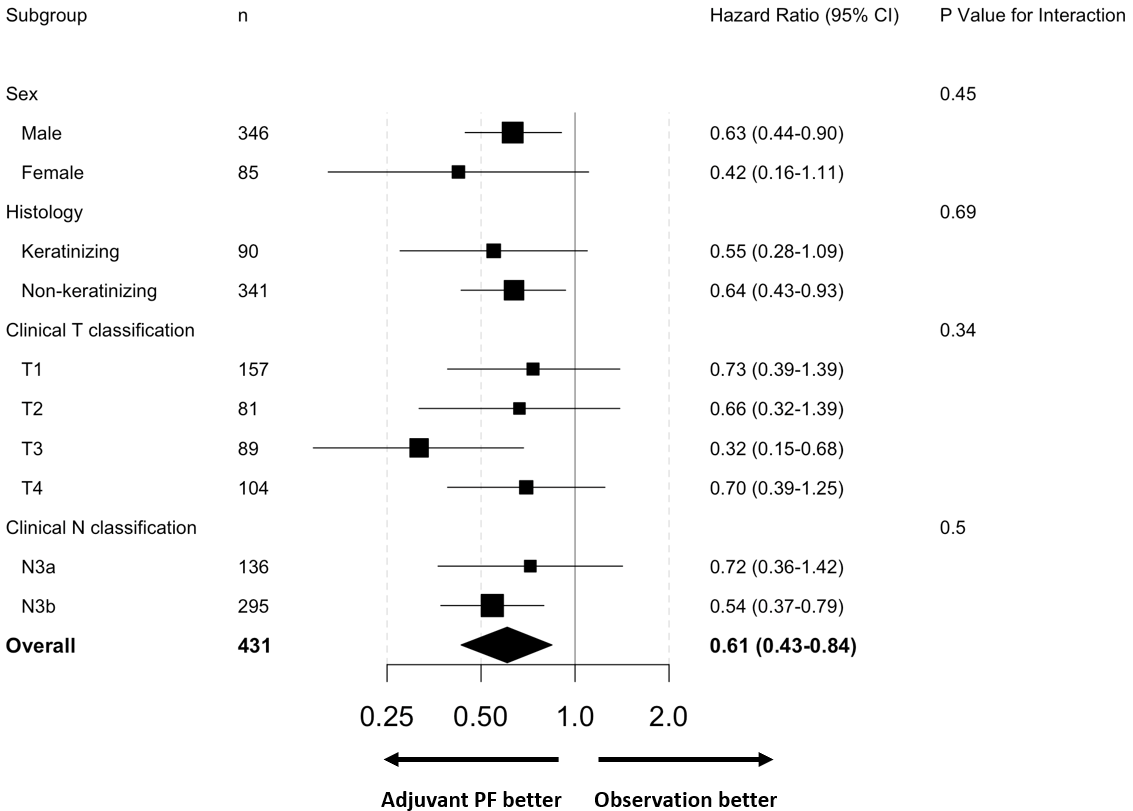


**Supplementary Figure 3** Kaplan-Meier survival curves of the propensity-matched cohort for overall survival show a higher survival rate in the adjuvant PF chemotherapy group. Figure created with R version 3.6.0 (https://www.r-project.org/).


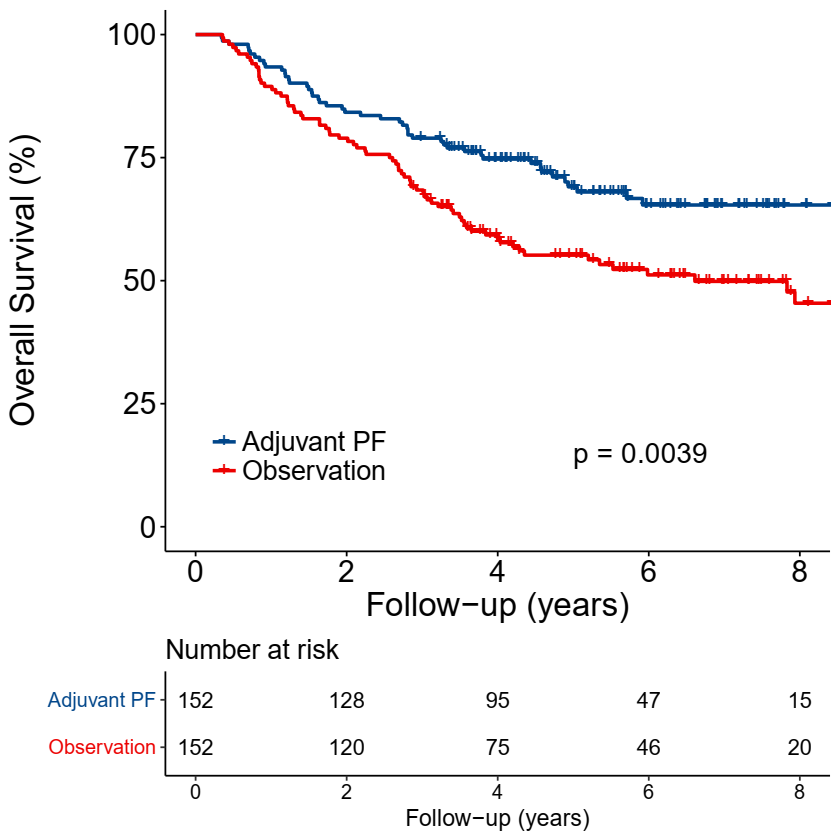


**Supplementary Figure 4** Landmark analyses restricted to patients surviving over (a) 12 months; (b) 18 months; (c) 24 months all show an improved survival in the adjuvant PF chemotherapy group, compatible with results of the primary analysis. Figure created with R version 3.6.0 (https://www.r-project.org/).


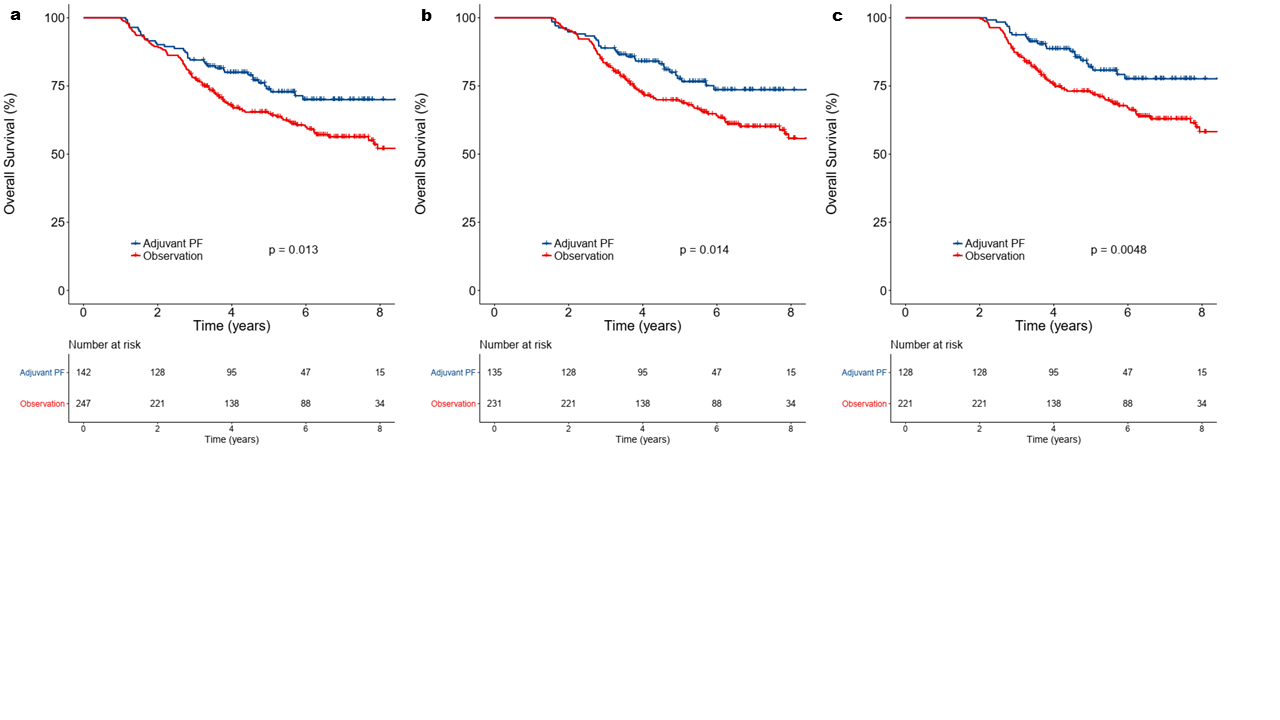


**Supplementary Table 1** Univariable and multivariable Cox proportional hazards model for distant metastasis-free survival (n = 310).

|  | **Univariable** |  |  | **Multivariable** |  |
| --- | --- | --- | --- | --- | --- |
| **Variable** | **Hazard ratio (95% CI)** | ***p*-value** |  | **Hazard ratio (95% CI)** | ***p*-value** |
| **Age, continuous** | 0.98 (0.95-1.02) | 0.32 |  | 0.97 (0.93-1.00) | 0.08 |
| **Sex** |  |  |  |  |  |
| Male | Reference |  |  | Reference |  |
| Female | 2.45 (1.06-5.67) | 0.04 |  | 2.16 (0.91-5.12) | 0.08 |
| **Histology^†^** |  |  |  |  |  |
| Lymphoepithelial / undifferentiated / NOS carcinoma | 0.57 (0.17-1.92) | 0.36 |  | 0.58 (0.16-2.07) | 0.40 |
| Squamous cell carcinoma, non-keratinizing | Reference |  |  | Reference |  |
| **Clinical T classification** |  |  |  |  |  |
| T1 | Reference |  |  | Reference |  |
| T2 | 2.04 (0.76-5.48) | 0.16 |  | 1.92 (0.71-5.24) | 0.20 |
| T3 | 1.30 (0.46-3.67) | 0.62 |  | 1.48 (0.52-4.23) | 0.46 |
| T4 | 0.23 (0.03-1.81) | 0.16 |  | 0.19 (0.02-1.55) | 0.12 |
| **Clinical N classification** |  |  |  |  |  |
| N3a | Reference |  |  | Reference |  |
| N3b | 0.93 (0.39-2.19) | 0.86 |  | 0.94 (0.38-2.30) | 0.89 |
| **Adjuvant treatment** |  |  |  |  |  |
| Observation | Reference |  |  | Reference |  |
| Adjuvant PF chemotherapy | 0.13 (0.03-0.54) | 0.005 |  | 0.11 (0.02-0.46) | 0.003 |

Abbreviations: CI, confidence interval; NOS, not otherwise specified

**^†^** There were no events for the *Squamous cell carcinoma, keratinizing or NOS* group

**Supplementary Table 2** Baseline patient and tumor characteristics of the propensity-matched cohort (n = 304).

| **Characteristics** | **Adjuvant PF Chemotherapy**  **(n = 152)** | **Observation**  **(n = 152)** | ***p*-value** |
| --- | --- | --- | --- |
| **Sex** |  |  | 0.75 |
| Male | 127 (83.6) | 130 (85.5) |  |
| Female | 25 (16.4) | 22 (14.5) |  |
| **Age, median (IQR)** | 46 (38.8-53) | 48 (38.8-56) | 0.15 |
| **Histology** |  |  | 0.47 |
| Lymphoepithelial / undifferentiated / NOS carcinoma / Squamous cell carcinoma, keratinizing or NOS | 41 (27.0) | 32 (21.1) |  |
| Squamous cell carcinoma, non-keratinizing | 111 (73.0) | 120 (78.9) |  |
| **Clinical T classification** |  |  | 0.99 |
| T1 | 51 (33.6) | 52 (34.2) |  |
| T2 | 30 (19.7) | 32 (21.1) |  |
| T3 | 34 (22.4) | 33 (21.7) |  |
| T4 | 37 (24.3) | 35 (23.0) |  |
| **Clinical N classification** |  |  | 0.14 |
| N3a | 42 (27.6) | 30 (19.7) |  |
| N3b | 110 (72.4) | 122 (80.3) |  |
| **Radiotherapy dose (Gray), median (IQR)** | 70 (70-72) | 72 (70-72) | 0.16 |
| **Radiotherapy fractions, median (IQR)** | 35 (35-37) | 36 (35-37) | 0.98 |

Abbreviations: IQR, interquartile range; NOS, not otherwise specified
